# Supplementary material for: Changes in risk perception and self-reported protective behaviour during the first week of the COVID-19 pandemic in the United States
Source: R Soc Open Sci. 2020 Sep 16;7(9):200742. doi: 10.1098/rsos.200742 (PMC7540790; doi:10.1098/rsos.200742)
Supplement: Supplementary material [file rsos200742supp1.pdf]

Supplementary results

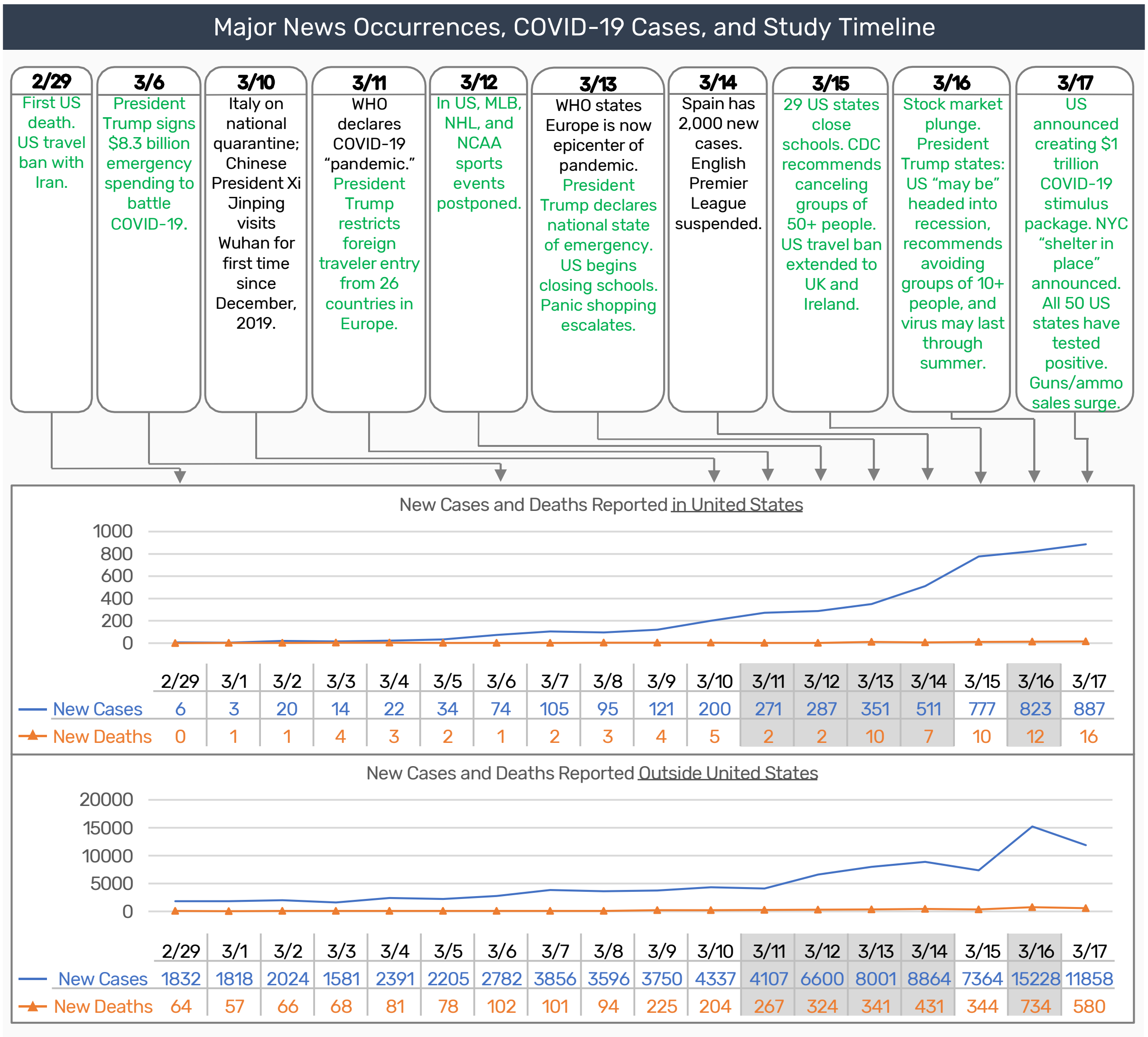

New Cases and Deaths Reported in United States

	2/29	3/1	3/2	3/3	3/4	3/5	3/6	3/7	3/8	3/9	3/10	3/11	3/12	3/13	3/14	3/15	3/16	3/17
New Cases	6	3	20	14	22	34	74	105	95	121	200	271	287	351	511	777	823	887
New Deaths	0	1	1	4	3	2	1	2	3	4	5	2	2	10	7	10	12	16

New Cases and Deaths Reported Outside United States

	2/29	3/1	3/2	3/3	3/4	3/5	3/6	3/7	3/8	3/9	3/10	3/11	3/12	3/13	3/14	3/15	3/16	3/17
New Cases	1832	1818	2024	1581	2391	2205	2782	3856	3596	3750	4337	4107	6600	8001	8864	7364	15228	11858
New Deaths	64	57	66	68	81	78	102	101	94	225	204	267	324	341	431	344	734	580

Figure S1. Timeline of events early in the United States COVID-19 pandemic. Days of current study data acquisition shown in gray. News events in green are most relevant for United States. COVID-19 data acquired from European Centre for Disease Prevention and Control (https://www.ecdc.europa.eu/en/publications-data/download-todays-data-geographic-distribution-covid-19-cases-worldwide). Major news events retrieved from National Broadcasting Company (NBC) News, Columbia Broadcasting System (CBS) News, and Cable News Network (CNN). Sources: https://www.cbsnews.com/live-updates/coronavirus-updates-cases-fears-deaths-us-latest-2020-03-16/; https://www.ecdc.europa.eu/en/geographical-distribution-2019-ncov-cases; https://www.nbcnews.com/health/health-news/coronavirus-timeline-tracking-critical-moments-covid-19-n1154341; https://www.cnn.com/2020/03/13/politics/donald-trump-emergency/index.html; https://www.cbsnews.com/live-updates/coronavirus-disease-covid-19-latest-news-2020-03-17/; https://www.cbsnews.com/live-updates/coronavirus-disease-covid-19-latest-news-2020-03-17/

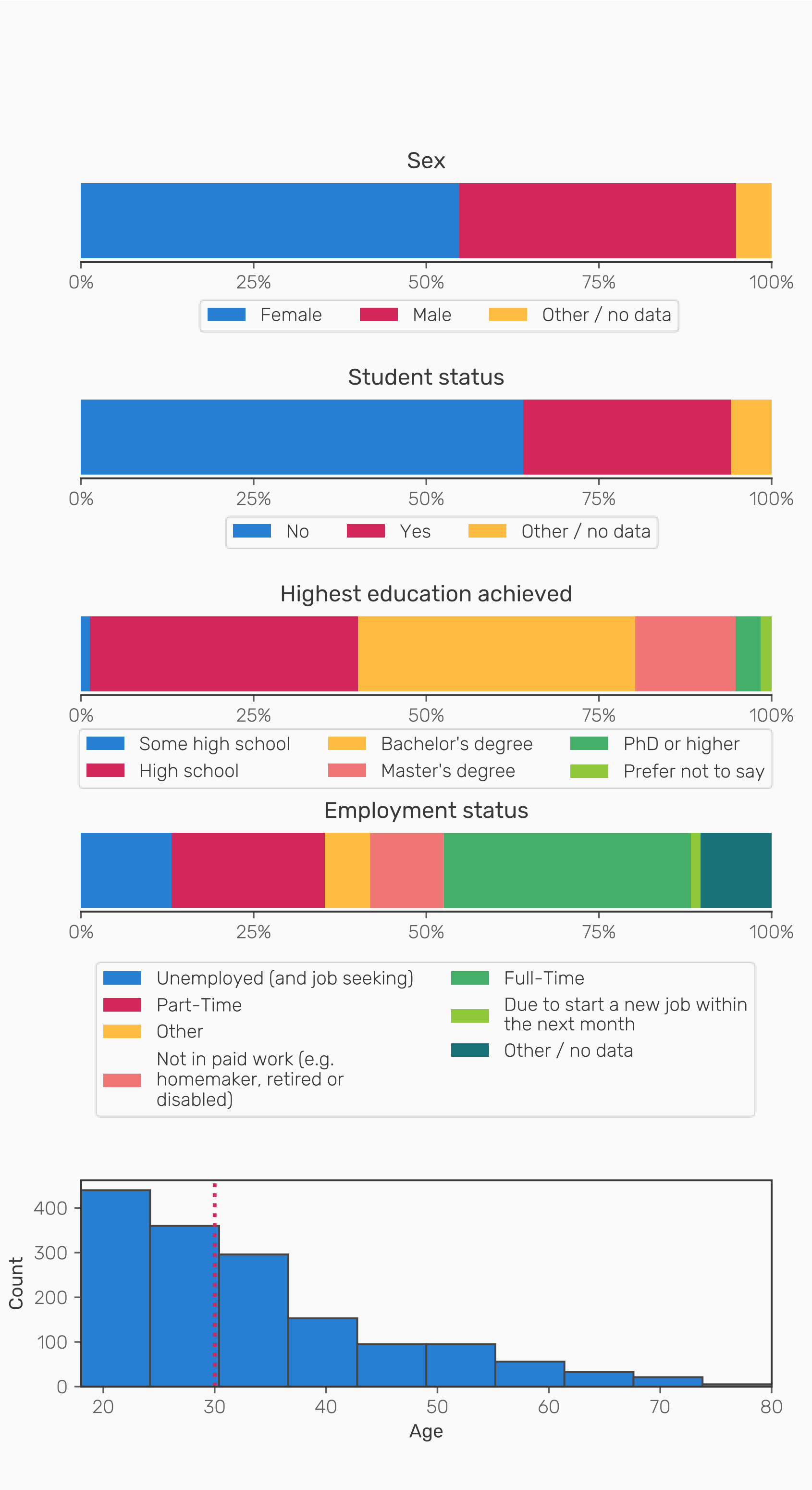

Figure S2. Demographics of survey participants. The red dotted line on the lower panel represents the median age (30 years).

**Question**	**df**	***F***	η_p^2	***p***
How likely do you think you are to catch the virus?	2, 1588	4.959	0.006	0.036
How badly do you think your health will be affected if you do catch the virus?	2, 1588	29.112	0.035	<.001
How badly do you think you will be affected economically if you specifically catch the virus (for example through loss of work)?	2, 1588	0.192	0	0.826
How badly do you think you will be affected by the global effects of the virus (for example economic recession, reduced healthcare capacity)?	2, 1588	0.315	0	0.811
How likely do you think it is that a loved one will become infected?	2, 1588	1.454	0.002	0.39
How likely do you think the average person in your neighborhood is to become infected?	2, 1588	3.997	0.005	0.062
How likely do you think the average person in your state is to become infected?	2, 1588	1.769	0.002	0.342
How likely do you think the average person in the USA is to become infected?	2, 1588	0.706	0.001	0.617
If you do contract the virus, how likely do you think it is that you will pass it on to someone else?	2, 1588	1.295	0.002	0.392
If you do contract the virus and pass it on to someone else, how badly do you think they would be affected?	2, 1588	2.389	0.003	0.23

Table S1. Effects of age on risk perception. Reported *p* values are FDR corrected.

Question	df	F	η_p^2	p
How likely would you be to attend an event with 10 other people?	2, 1588	11.6	0.014	<.001
How likely would you be to attend an event with 50 other people?	2, 1588	4.596	0.006	0.031
How likely would you be to attend an event with 100 other people?	2, 1588	2.948	0.004	0.127
How likely would you be to attend an event with 500 other people?	2, 1588	1.792	0.002	0.224
How likely would you be to attend an event with 1000 other people?	2, 1588	0.566	0.001	0.62
I avoid watching/reading the news about the virus because it makes me worried.	2, 1588	9.783	0.012	<.001
The virus has caused me to avoid in-person social interactions (e.g., friends, family, co-workers, strangers).	2, 1588	10.726	0.013	<.001
I am buying more sanitary products (e.g. soap, hand sanitizer) than I normally would	2, 1588	1.768	0.002	0.224
I am buying more food and water than I would normally	2, 1588	1.681	0.002	0.224
I am washing my hands more than I would normally	2, 1588	0.436	0.001	0.646
I am staying home more than I normally would	2, 1588	1.96	0.002	0.224
I am travelling less than I would normally	2, 1588	2.08	0.003	0.224

Table S2. Effects of age on protective behaviors. Reported *p* values are FDR corrected.

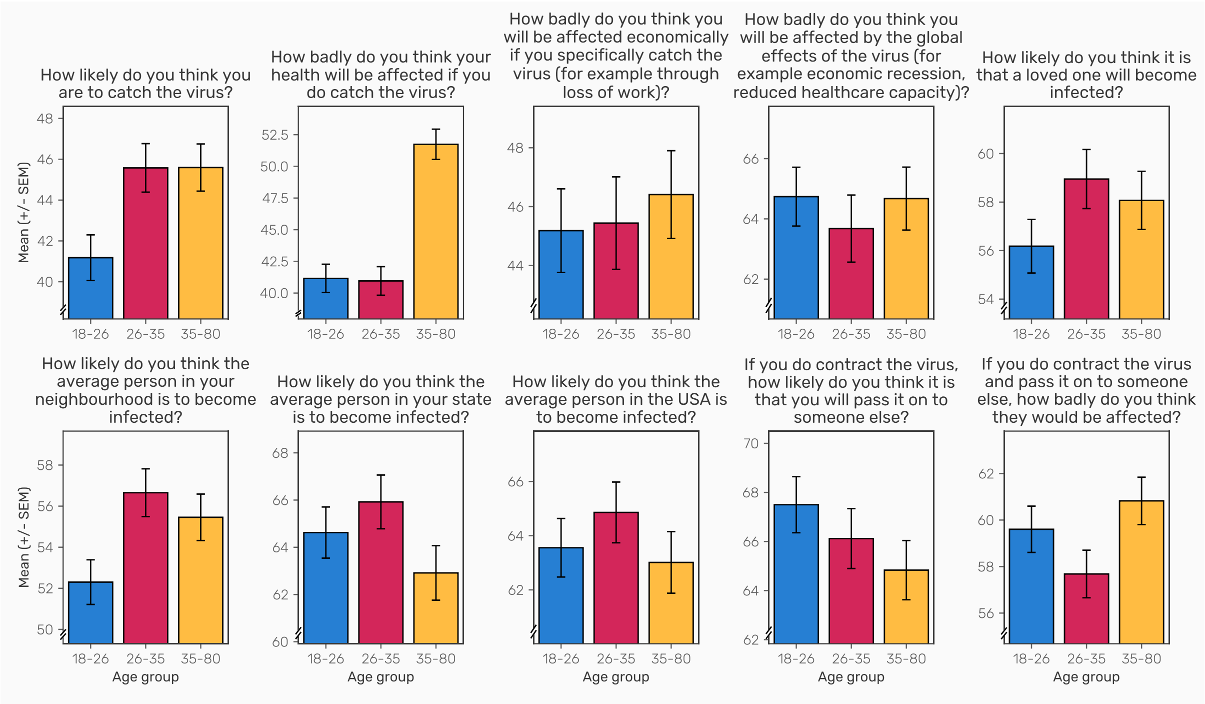

Figure S3. Effects of age on risk perception. Note: Y axes are restricted to display differences between groups.

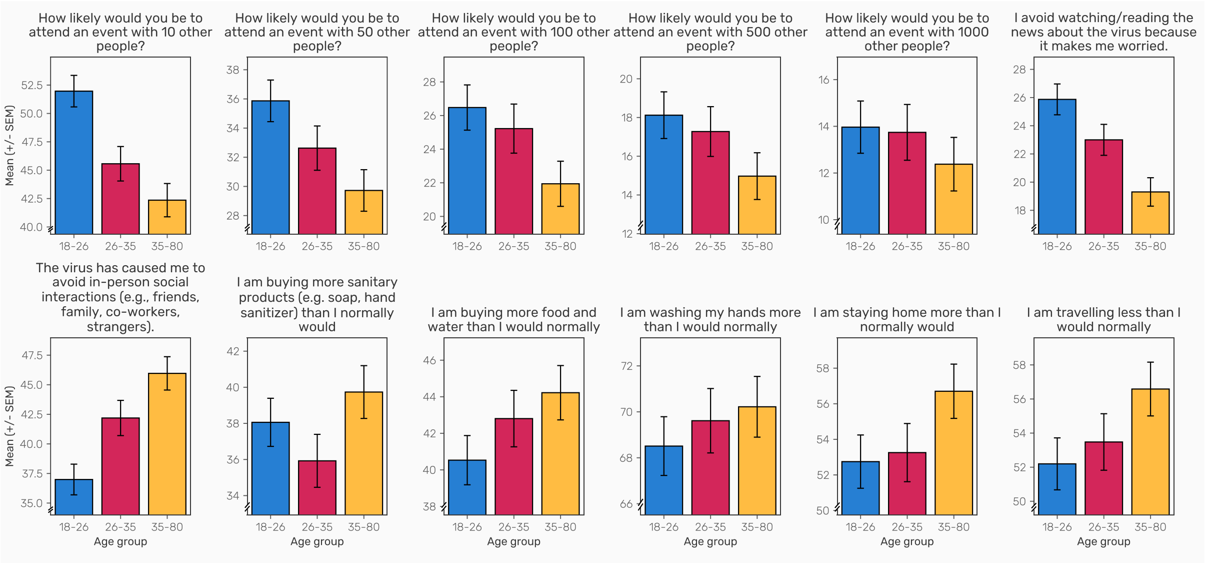

Figure S4. Effects of age on protective behaviors. Note: Y axes are restricted to display differences between groups.

Question	df	*t*	*p*
How likely do you think you are to catch the virus?	1511	-0.41	0.754
How badly do you think your health will be affected if you do catch the virus?	1511	0.86	0.62
How badly do you think you will be affected economically if you specifically catch the virus (for example through loss of work)?	1511	0.56	0.716
How badly do you think you will be affected by the global effects of the virus (for example economic recession, reduced healthcare capacity)?	1511	0.78	0.62
How likely do you think it is that a loved one will become infected?	1511	-0.02	0.985
How likely do you think the average person in your neighbourhood is to become infected?	1511	-0.87	0.62
How likely do you think the average person in your state is to become infected?	1511	1.12	0.62
How likely do you think the average person in the USA is to become infected?	1511	1.76	0.264
If you do contract the virus, how likely do you think it is that you will pass it on to someone else?	1511	4.57	< .001
If you do contract the virus and pass it on to someone else, how badly do you think they would be affected?	1511	2.02	0.22

Table S3. Effects of sex on risk perception. Reported *p* values are FDR corrected.

Question	df	*t*	*p*
How likely would you be to attend an event with 10 other people?	1511	-0.25	0.817
How likely would you be to attend an event with 50 other people?	1511	-0.71	0.639
How likely would you be to attend an event with 100 other people?	1511	-1.01	0.537
How likely would you be to attend an event with 500 other people?	1511	-1.6	0.326
How likely would you be to attend an event with 1000 other people?	1511	-1.45	0.356
I avoid watching/reading the news about the virus because it makes me worried.	1511	2.84	0.047
The virus has caused me to avoid in-person social interactions (e.g., friends, family, co-workers, strangers).	1511	2.66	0.047
I am buying more sanitary products (e.g. soap, hand sanitizer) than I normally would	1511	-0.88	0.569
I am buying more food and water than I would normally	1511	1.06	0.537
I am washing my hands more than I would normally	1511	1.62	0.326
I am staying home more than I normally would	1511	-0.23	0.817
I am travelling less than I would normally	1511	-0.43	0.804

Table S4. Effects of sex on protective behaviors. Reported *p* values are FDR corrected

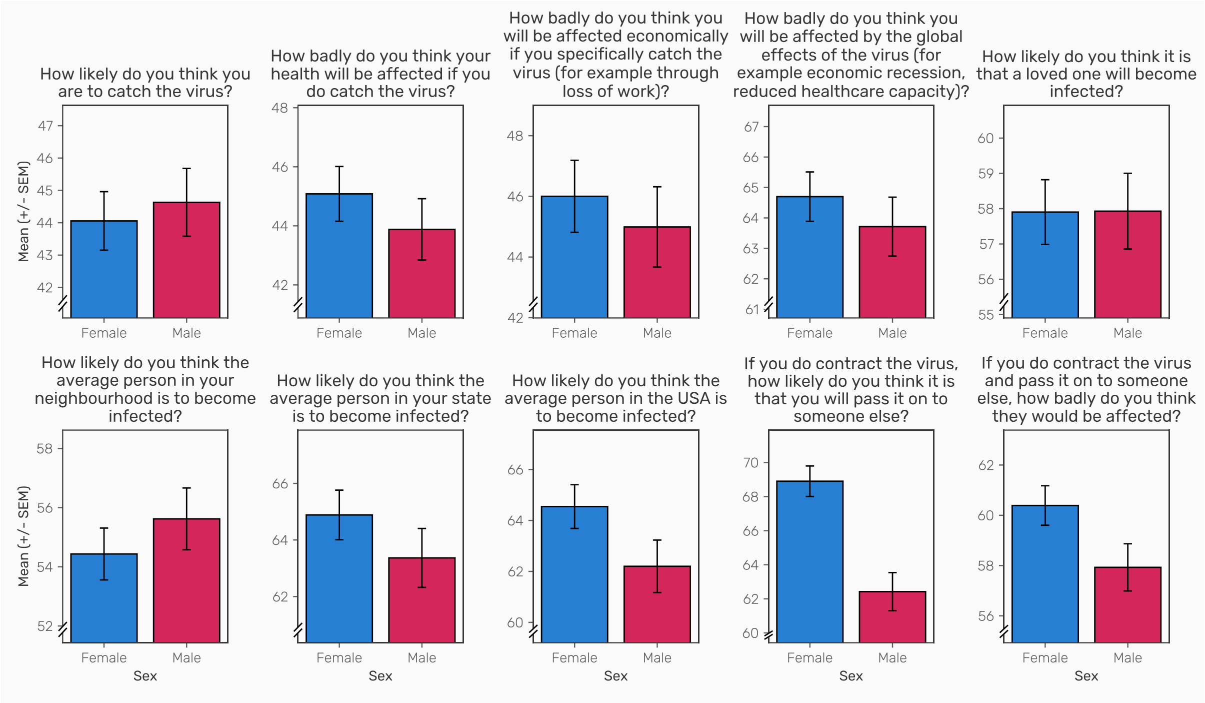

Figure S5. Effects of sex on risk perception. Note: Y axes are restricted to display differences between groups.

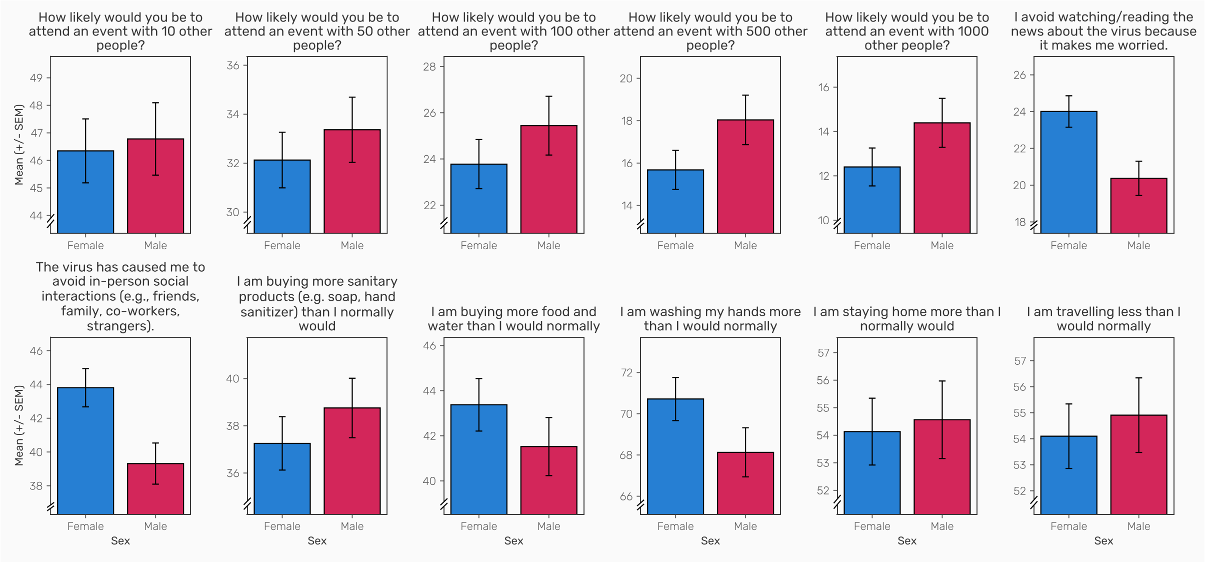

Figure S6. Effects of sex on protective behaviors. Note: Y axes are restricted to display differences between groups.

**Question**	**df**	***F***	η_p^2	***p***
How likely do you think you are to catch the virus?	3, 942	2.32	0.007	0.148
How badly do you think your health will be affected if you do catch the virus?	3, 942	1.093	0.003	0.39
How badly do you think you will be affected economically if you specifically catch the virus (for example through loss of work)?	3, 942	2.101	0.007	0.164
How badly do you think you will be affected by the global effects of the virus (for example economic recession, reduced healthcare capacity)?	3, 942	2.322	0.007	0.148
How likely do you think it is that a loved one will become infected?	3, 942	1.372	0.004	0.312
How likely do you think the average person in your neighbourhood is to become infected?	3, 942	3.185	0.01	0.093
How likely do you think the average person in your state is to become infected?	3, 942	1.892	0.006	0.185
How likely do you think the average person in the USA is to become infected?	3, 942	0.995	0.003	0.395
If you do contract the virus, how likely do you think it is that you will pass it on to someone else?	3, 942	3.046	0.01	0.093
If you do contract the virus and pass it on to someone else, how badly do you think they would be affected?	3, 942	3.354	0.011	0.093

Table S5. Effects of location on risk perception. Reported *p* values are FDR corrected

**Question**	**df**	***F***	η_p^2	***p***
How likely would you be to attend an event with 10 other people?	3, 942	3.798	0.012	0.02
How likely would you be to attend an event with 50 other people?	3, 942	2.42	0.008	0.097
How likely would you be to attend an event with 100 other people?	3, 942	1.693	0.005	0.222
How likely would you be to attend an event with 500 other people?	3, 942	0.937	0.003	0.507
How likely would you be to attend an event with 1000 other people?	3, 942	0.452	0.001	0.781
I avoid watching/reading the news about the virus because it makes me worried.	3, 942	0.303	0.001	0.824
The virus has caused me to avoid in-person social interactions (e.g., friends, family, co-workers, strangers).	3, 942	8.576	0.027	< .001
I am buying more sanitary products (e.g. soap, hand sanitizer) than I normally would	3, 942	4.931	0.015	0.006
I am buying more food and water than I would normally	3, 942	4.651	0.015	0.007
I am washing my hands more than I would normally	3, 942	3.062	0.01	0.047
I am staying home more than I normally would	3, 942	11.068	0.034	< .001
I am travelling less than I would normally	3, 942	7.676	0.024	< .001

Table S6. Effects of location on protective behaviors. Reported *p* values are FDR corrected

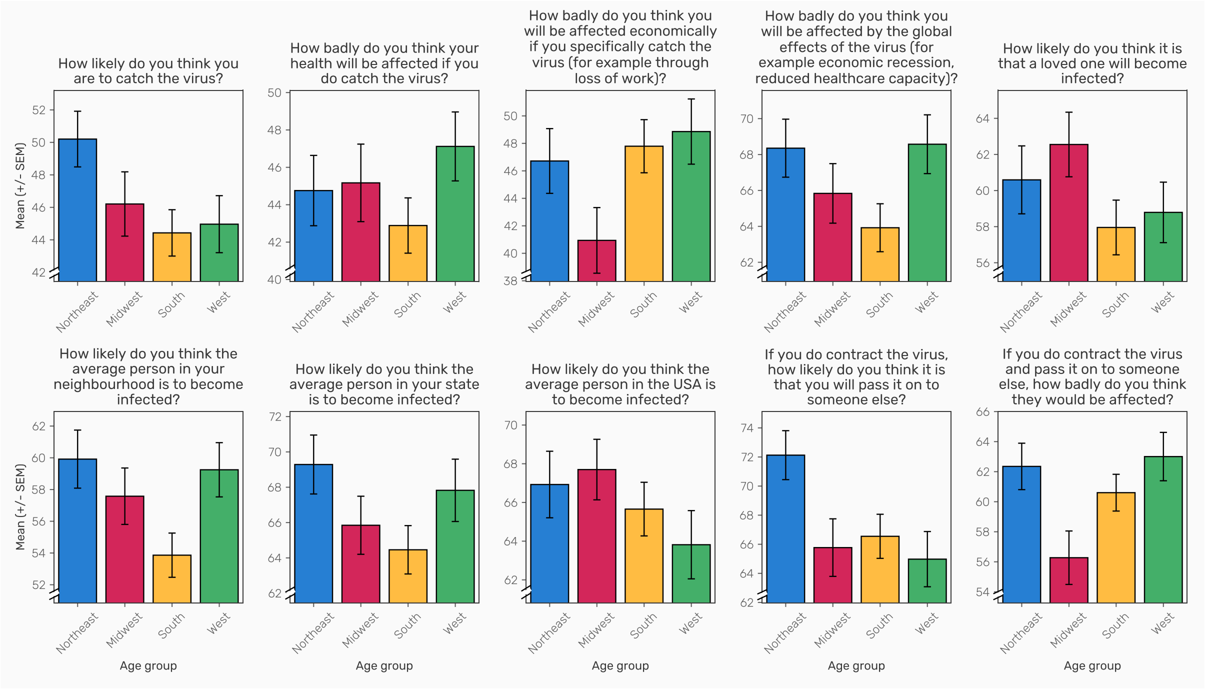

Figure S7. Effects of location on risk perception. Note: Y axes are restricted to facilitate comparisons between groups.

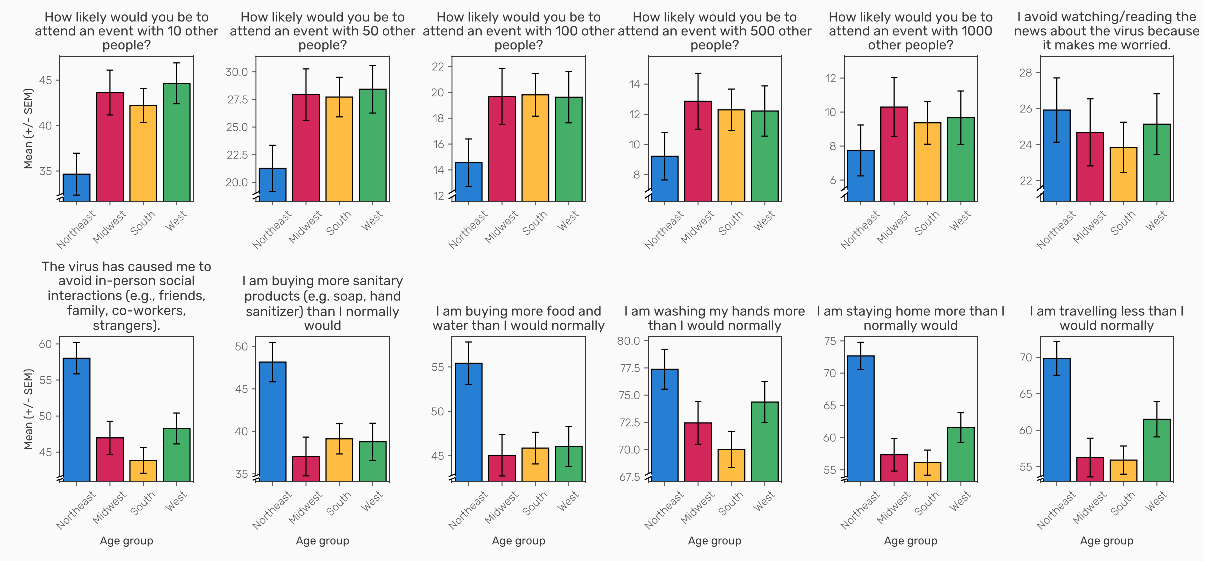

Figure S8. Effects of location on risk perception. Note: Y axes are restricted to facilitate comparisons between groups.

Question	df	*t*	*p*
How likely do you think you are to catch the virus?	1266	4.85	<.001
How badly do you think your health will be affected if you do catch the virus?	1266	-4.04	<.001
How badly do you think you will be affected economically if you specifically catch the virus (for example through loss of work)?	1266	-0.18	0.854
How badly do you think you will be affected by the global effects of the virus (for example economic recession, reduced healthcare capacity)?	1266	1.93	0.09
How likely do you think it is that a loved one will become infected?	1266	2.81	0.012
How likely do you think the average person in your neighbourhood is to become infected?	1266	4.73	<.001
How likely do you think the average person in your state is to become infected?	1266	1.7	0.126
How likely do you think the average person in the USA is to become infected?	1266	0.35	0.811
If you do contract the virus, how likely do you think it is that you will pass it on to someone else?	1266	0.7	0.608
If you do contract the virus and pass it on to someone else, how badly do you think they would be affected?	1266	-2.21	0.054

Table S7. Effects of education level on risk perception. Results reflect the t values for the effect of education level in a regression model controlling for age. Reported *p* values are FDR corrected

**Question**	**df**	***t***	***p***
How likely would you be to attend an event with 10 other people?	1266	-4.82	<.001
How likely would you be to attend an event with 50 other people?	1266	-5.73	<.001
How likely would you be to attend an event with 100 other people?	1266	-5.84	<.001
How likely would you be to attend an event with 500 other people?	1266	-5.94	<.001
How likely would you be to attend an event with 1000 other people?	1266	-5.54	<.001
I avoid watching/reading the news about the virus because it makes me worried.	1266	1.69	0.091
The virus has caused me to avoid in-person social interactions (e.g., friends, family, co-workers, strangers).	1266	7.93	<.001
I am buying more sanitary products (e.g. soap, hand sanitizer) than I normally would	1266	3.89	<.001
I am buying more food and water than I would normally	1266	6.79	<.001
I am washing my hands more than I would normally	1266	5.90	<.001
I am staying home more than I normally would	1266	8.73	<.001
I am travelling less than I would normally	1266	8.59	<.001

Table S8. Effects of education level on protective behaviors. Results reflect the t values for the effect of education level in a regression model controlling for age. Reported *p* values are FDR corrected

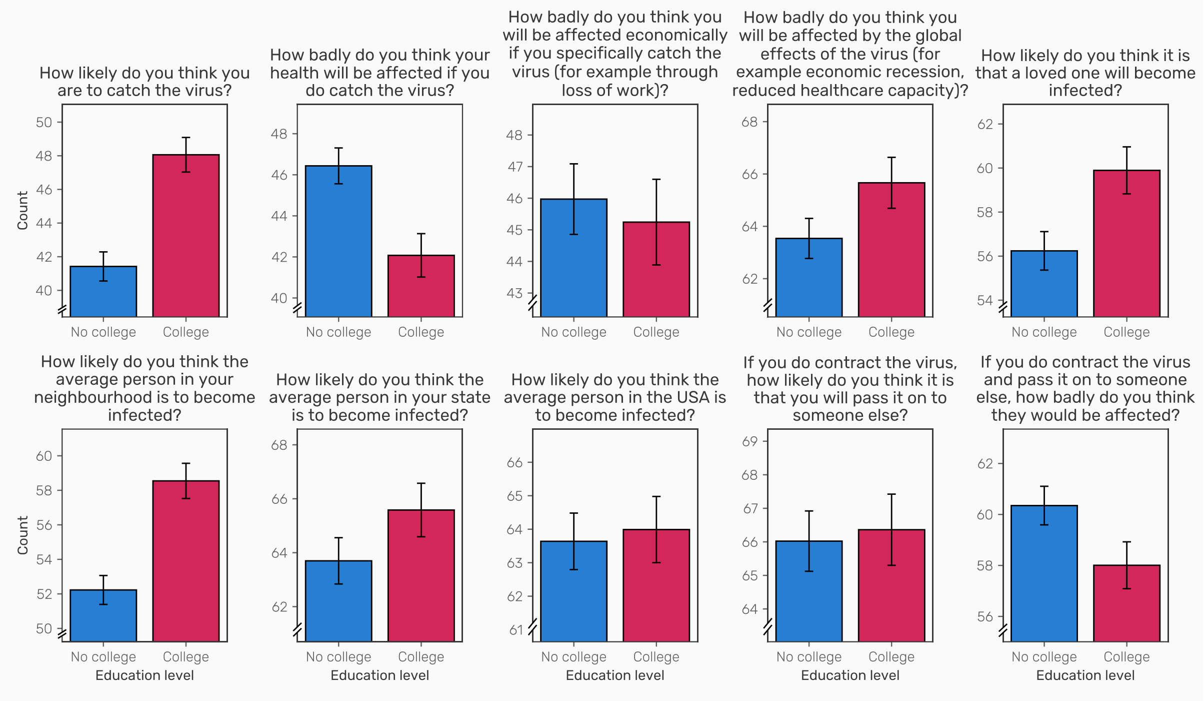

Figure S9. Effects of education level on risk perception. Note: Y axes are restricted to facilitate comparisons between groups.

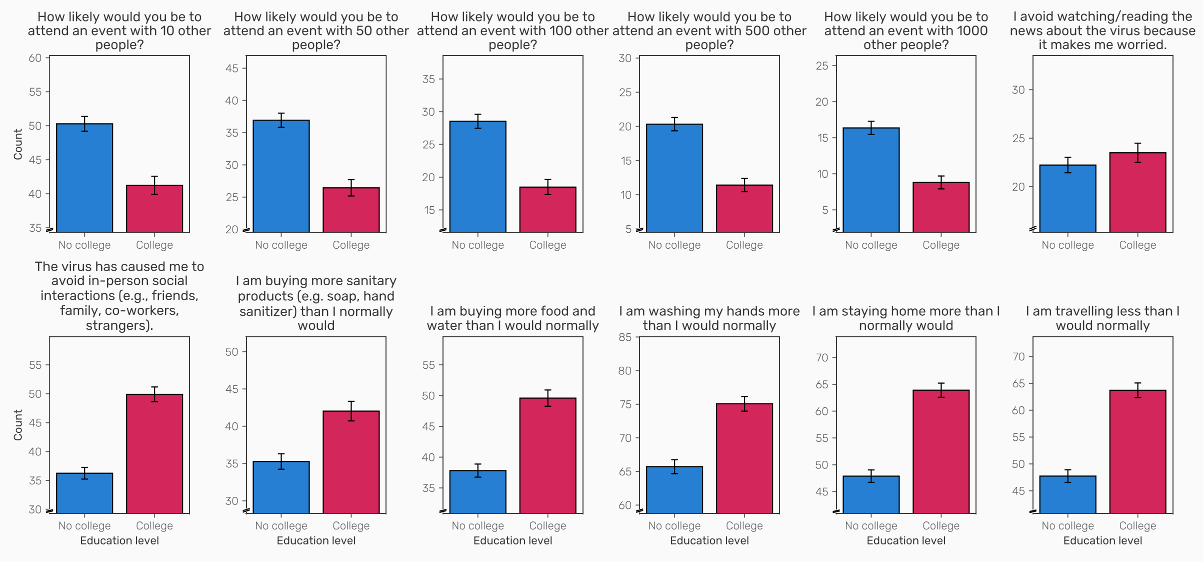

Figure S10. Effects of education level on protective behaviors. Note: Y axes are restricted to facilitate comparisons between groups.

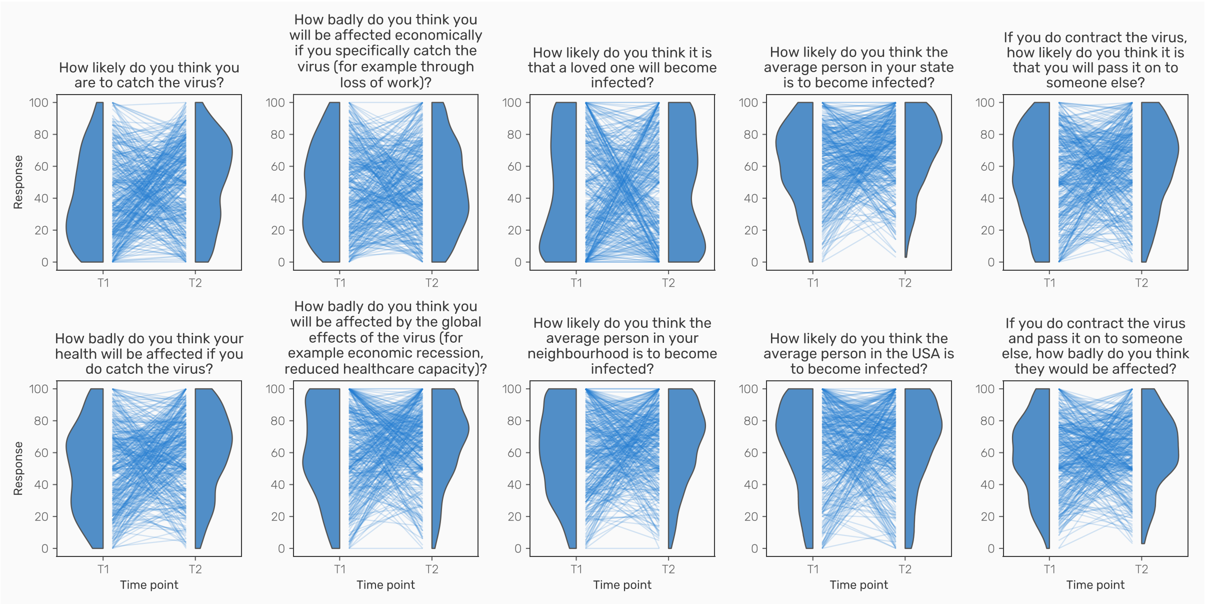

Figure S9. Within-subject changes in responses to questions about risk perception between the two timepoints.

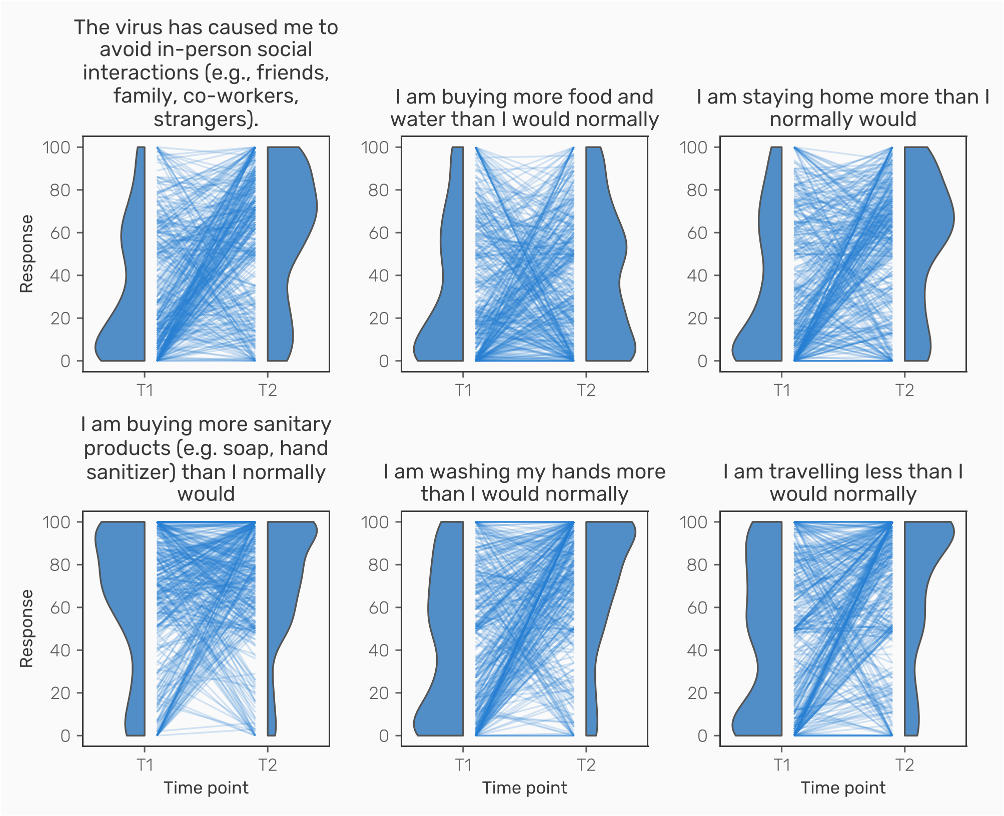

Figure S10. Within-subject changes in responses to questions about engagement in protective behaviour between the two timepoints.

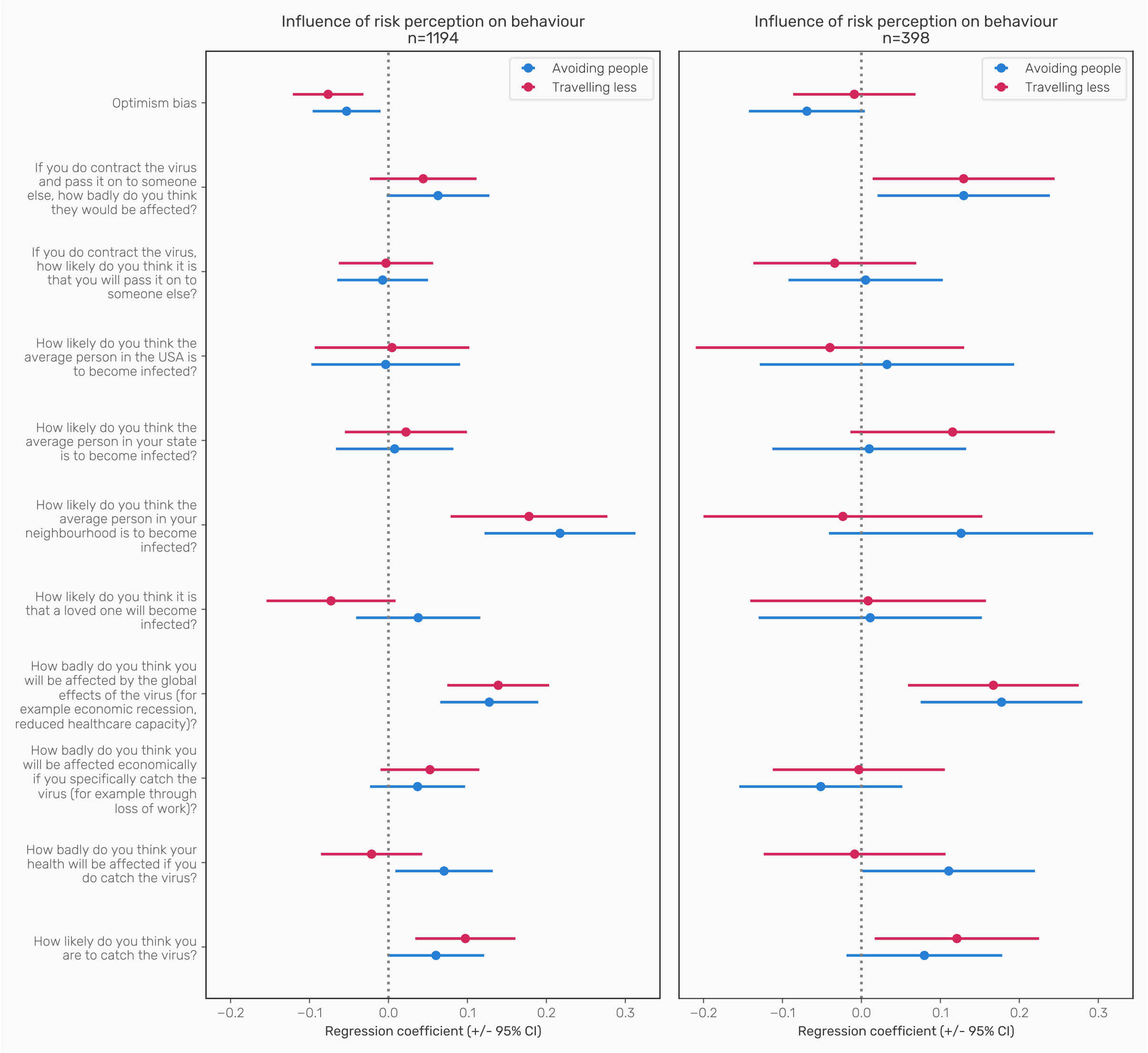

Figure S11. Results of regression analyses using avoidance of social interactions and reduction in travelling less as dependent variables.

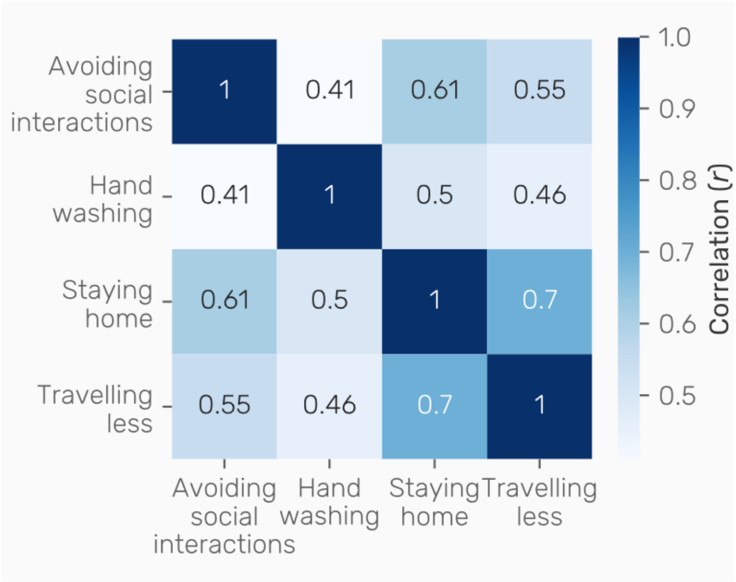

Figure S12. Correlations between measures of engagement in protective behaviors.

Table S7. Results of multiple regression predicting engagement in hand washing and social distancing from measures of risk perception in the discovery sample (75% of the data). *** = p <.001, ** = p < .01, * = p < .05

**Outcome**	**Predictor**	***β***	***β* 95% CI**	***p***
Hand washing	Intercept	0	-0.05, 0.06	0.943
	Age	0.03	-0.02, 0.09	0.266
	How likely do you think you are to catch the virus?	0.17***	0.09, 0.25	<.001
	How badly do you think your health will be affected if you do catch the virus?	-0.03	-0.09, 0.03	0.367
	How badly do you think you will be affected economically if you specifically catch the virus (for example through loss of work)?	0.02	-0.04, 0.09	0.46
	How badly do you think you will be affected by the global effects of the virus (for example economic recession, reduced healthcare capacity)?	0.08*	0.02, 0.15	0.012
	How likely do you think it is that a loved one will become infected?	-0.01	-0.09, 0.07	0.846
	How likely do you think the average person in your neighbourhood is to become infected?	0.12*	0.02, 0.22	0.016
	How likely do you think the average person in your state is to become infected?	0	-0.1, 0.11	0.97
	How likely do you think the average person in the USA is to become infected?	-0.01	-0.1, 0.09	0.906
	If you do contract the virus, how likely do you think it is that you will pass it on to someone else?	0.05	-0.01, 0.11	0.073
	If you do contract the virus and pass it on to someone else, how badly do you think they would be affected?	0.04	-0.02, 0.11	0.208
Social distancing	Intercept	0	-0.05, 0.05	0.99
	Age	0.02	-0.03, 0.08	0.431
	How likely do you think you are to catch the virus?	0.2***	0.12, 0.28	<.001
	How badly do you think your health will be affected if you do catch the virus?	0	-0.06, 0.07	0.948
	How badly do you think you will be affected economically if you specifically catch the virus (for example through loss of work)?	0.04	-0.02, 0.1	0.187
	How badly do you think you will be affected by the global effects of the virus (for example economic recession, reduced healthcare capacity)?	0.14***	0.08, 0.2	<.001
	How likely do you think it is that a loved one will become infected?	-0.01	-0.09, 0.07	0.736
	How likely do you think the average person in your neighbourhood is to become infected?	0.16***	0.06, 0.26	0.001
	How likely do you think the average person in your state is to become infected?	-0.01	-0.12, 0.09	0.782
	How likely do you think the average person in the USA is to become infected?	-0.05	-0.15, 0.05	0.299
	If you do contract the virus, how likely do you think it is that you will pass it on to someone else?	-0.03	-0.09, 0.03	0.374
	If you do contract the virus and pass it on to someone else, how badly do you think they would be affected?	0.04	-0.03, 0.11	0.26

Table S8. Results of multiple regression predicting engagement in hand washing and social distancing from measures of risk perception in the validation sample (25% of the data). *** = p <.001, ** = p < .01, * = p < .05

**Outcome**	**Predictor**	***β***	***B* 95% CI**	***p***
Hand washing	Intercept	-0.01	-0.11, 0.09	0.871
	Age	0.02	-0.08, 0.12	0.72
	How likely do you think you are to catch the virus?	0.2***	0.07, 0.34	0.004
	How badly do you think your health will be affected if you do catch the virus?	-0.03	-0.15, 0.08	0.567
	How badly do you think you will be affected economically if you specifically catch the virus (for example through loss of work)?	0.04	-0.07, 0.16	0.461
	How badly do you think you will be affected by the global effects of the virus (for example economic recession, reduced healthcare capacity)?	0.13*	0.02, 0.24	0.017
	How likely do you think it is that a loved one will become infected?	-0.13	-0.29, 0.03	0.102
	How likely do you think the average person in your neighbourhood is to become infected?	0	-0.18, 0.19	0.96
	How likely do you think the average person in your state is to become infected?	0.1	-0.09, 0.29	0.297
	How likely do you think the average person in the USA is to become infected?	-0.05	-0.22, 0.12	0.547
	If you do contract the virus, how likely do you think it is that you will pass it on to someone else?	0.07	-0.04, 0.17	0.211
	If you do contract the virus and pass it on to someone else, how badly do you think they would be affected?	0.01	-0.11, 0.12	0.921
Social distancing	Intercept	0.01	-0.08, 0.11	0.822
	Age	0.01	-0.09, 0.11	0.804
	How likely do you think you are to catch the virus?	0.17*	0.04, 0.3	0.011
	How badly do you think your health will be affected if you do catch the virus?	0.03	-0.08, 0.14	0.618
	How badly do you think you will be affected economically if you specifically catch the virus (for example through loss of work)?	0.12*	0.0, 0.23	0.041
	How badly do you think you will be affected by the global effects of the virus (for example economic recession, reduced healthcare capacity)?	0.11*	0.0, 0.21	0.046
	How likely do you think it is that a loved one will become infected?	-0.02	-0.17, 0.14	0.844
	How likely do you think the average person in your neighbourhood is to become infected?	0.13	-0.05, 0.3	0.16
	How likely do you think the average person in your state is to become infected?	0.11	-0.08, 0.29	0.256
	How likely do you think the average person in the USA is to become infected?	-0.15	-0.31, 0.02	0.079
	If you do contract the virus, how likely do you think it is that you will pass it on to someone else?	-0.01	-0.12, 0.09	0.777
	If you do contract the virus and pass it on to someone else, how badly do you think they would be affected?	0.06	-0.06, 0.17	0.327

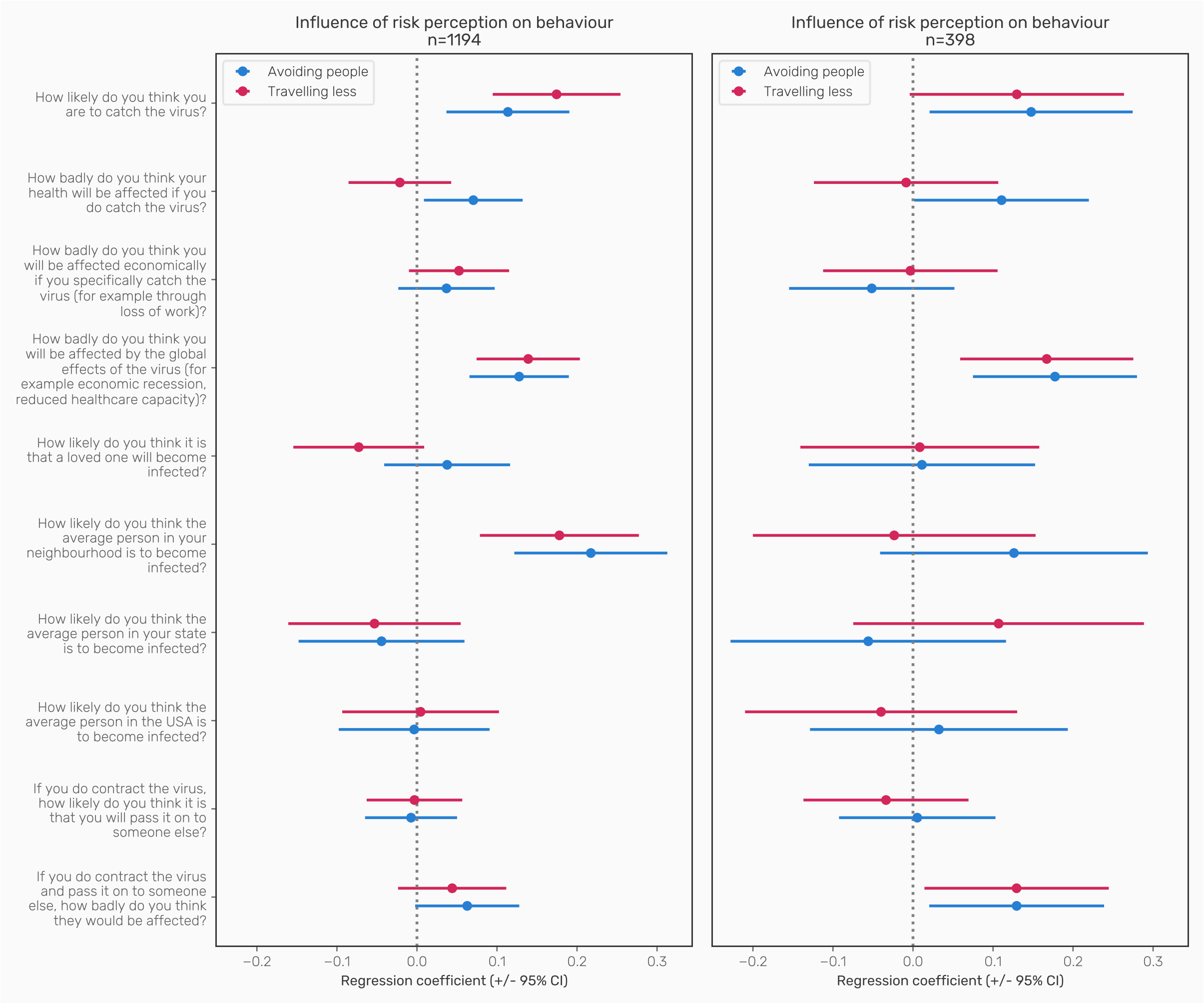

Figure S13. Results of regression analyses using avoidance of social interactions and reduction in travelling less as dependent variables.

Table S9. Results of multiple regression predicting engagement in avoidance of social situations and reductions in travel from measures of risk perception in the discovery sample (75% of the data). *** = p <.001, ** = p < .01, * = p < .05

Outcome	Predictor	*β*	*B* 95% CI	*p*
Avoiding people	Intercept	0	-0.05, 0.06	0.925
	Age	0.05	-0.0, 0.11	0.052
	How likely do you think you are to catch the virus?	0.11	0.04, 0.19	0.004
	How badly do you think your health will be affected if you do catch the virus?	0.07	0.01, 0.13	0.025
	How badly do you think you will be affected economically if you specifically catch the virus (for example through loss of work)?	0.04	-0.02, 0.1	0.23
	How badly do you think you will be affected by the global effects of the virus (for example economic recession, reduced healthcare capacity)?	0.13	0.07, 0.19	0
	How likely do you think it is that a loved one will become infected?	0.04	-0.04, 0.12	0.347
	How likely do you think the average person in your neighborhood is to become infected?	0.22	0.12, 0.31	0
	How likely do you think the average person in your state is to become infected?	-0.04	-0.15, 0.06	0.402
	How likely do you think the average person in the USA is to become infected?	0	-0.1, 0.09	0.942
	If you do contract the virus, how likely do you think it is that you will pass it on to someone else?	-0.01	-0.06, 0.05	0.801
	If you do contract the virus and pass it on to someone else, how badly do you think they would be affected?	0.06	-0.0, 0.13	0.059
Travelling less	Intercept	0	-0.05, 0.06	0.881
	Age	0.05	-0.01, 0.1	0.114
	How likely do you think you are to catch the virus?	0.17	0.09, 0.25	0
	How badly do you think your health will be affected if you do catch the virus?	-0.02	-0.09, 0.04	0.513
	How badly do you think you will be affected economically if you specifically catch the virus (for example through loss of work)?	0.05	-0.01, 0.12	0.1
	How badly do you think you will be affected by the global effects of the virus (for example economic recession, reduced healthcare capacity)?	0.14	0.07, 0.2	0
	How likely do you think it is that a loved one will become infected?	-0.07	-0.15, 0.01	0.081
	How likely do you think the average person in your neighborhood is to become infected?	0.18	0.08, 0.28	0
	How likely do you think the average person in your state is to become infected?	-0.05	-0.16, 0.05	0.334
	How likely do you think the average person in the USA is to become infected?	0	-0.09, 0.1	0.928
	If you do contract the virus, how likely do you think it is that you will pass it on to someone else?	0	-0.06, 0.06	0.919
	If you do contract the virus and pass it on to someone else, how badly do you think they would be affected?	0.04	-0.02, 0.11	0.202

Table S10. Results of multiple regression predicting engagement in avoidance of social situations and reductions in travel from measures of risk perception in the validation sample (25% of the data). *** = p <.001, ** = p < .01, * = p < .05

Outcome	Predictor	*β*	*B* 95% CI	*p*
Avoiding people	Intercept	0.01	-0.08, 0.1	0.901
	Age	0.06	-0.04, 0.15	0.22
	How likely do you think you are to catch the virus?	0.15	0.02, 0.27	0.023
	How badly do you think your health will be affected if you do catch the virus?	0.11	0.0, 0.22	0.047
	How badly do you think you will be affected economically if you specifically catch the virus (for example through loss of work)?	-0.05	-0.15, 0.05	0.327
	How badly do you think you will be affected by the global effects of the virus (for example economic recession, reduced healthcare capacity)?	0.18	0.07, 0.28	0.001
	How likely do you think it is that a loved one will become infected?	0.01	-0.13, 0.15	0.877
	How likely do you think the average person in your neighborhood is to become infected?	0.13	-0.04, 0.29	0.139
	How likely do you think the average person in your state is to become infected?	-0.06	-0.23, 0.12	0.523
	How likely do you think the average person in the USA is to become infected?	0.03	-0.13, 0.19	0.693
	If you do contract the virus, how likely do you think it is that you will pass it on to someone else?	0.01	-0.09, 0.1	0.914
	If you do contract the virus and pass it on to someone else, how badly do you think they would be affected?	0.13	0.02, 0.24	0.02
Travelling less	Intercept	0	-0.1, 0.1	0.994
	Age	0.01	-0.09, 0.11	0.787
	How likely do you think you are to catch the virus?	0.13	-0.0, 0.26	0.057
	How badly do you think your health will be affected if you do catch the virus?	-0.01	-0.12, 0.11	0.884
	How badly do you think you will be affected economically if you specifically catch the virus (for example through loss of work)?	0	-0.11, 0.11	0.953
	How badly do you think you will be affected by the global effects of the virus (for example economic recession, reduced healthcare capacity)?	0.17	0.06, 0.28	0.003
	How likely do you think it is that a loved one will become infected?	0.01	-0.14, 0.16	0.911
	How likely do you think the average person in your neighborhood is to become infected?	-0.02	-0.2, 0.15	0.794
	How likely do you think the average person in your state is to become infected?	0.11	-0.07, 0.29	0.248
	How likely do you think the average person in the USA is to become infected?	-0.04	-0.21, 0.13	0.645
	If you do contract the virus, how likely do you think it is that you will pass it on to someone else?	-0.03	-0.14, 0.07	0.521
	If you do contract the virus and pass it on to someone else, how badly do you think they would be affected?	0.13	0.01, 0.24	0.028

Table S11. Results of logistic regression predicting membership of the low engagement group from demographic variables, measures of risk perception and reported frequency of searching online for information. *** = p <.001, ** = p < .01, * = p < .05

**Predictor**	***β***	***B* 95% CI**	***p***
Intercept	-2.87	-3.26, -2.49	<.001
Sex	-0.15	-0.69, 0.38	0.577
Age	-0.12	-0.43, 0.2	0.467
Personal likelihood	-0.27	-0.6, 0.05	0.098
Personal severity	-0.34	-0.65, -0.02	0.04
Economic effects	0.08	-0.19, 0.36	0.552
Average likelihood	0.23	-0.08, 0.53	0.143
Transmission likelihood	0.06	-0.21, 0.33	0.649
Transmission severity	0.03	-0.27, 0.33	0.849
Searching online	-0.31	-0.57, -0.05	0.018
Personally affected	-0.81	-1.14, -0.49	<.001
Avoiding news	0.03	-0.25, 0.31	0.827
Education level	-0.3	-0.62, 0.03	0.074
